# Editorial: The second international workshop on health natural language processing (HealthNLP 2019)

**DOI:** 10.1186/s12911-019-0930-9

**Published:** 2019-12-05

**Authors:** Yanshan Wang, Hua Xu, Ozlem Uzuner

**Affiliations:** 1 Department of Health Sciences Research, Mayo Clinic, Rochester, MN USA; 2 School of Biomedical Informatics, The University of Texas Health Science Center at Houston, Houston, TX USA; 3 Information Sciences and Technology, George Mason University, Fairfax, VA USA

**Keywords:** Natural language processing, NLP, Healthcare, Electronic health records, EHR, Artificial intelligence

## Background

In the past few decades, growing adoption of electronic health record (EHR) systems has made massive clinical narrative data available electronically. Natural language processing (NLP) technologies that can unlock information from narrative text have received great attention in the medical domain. Many clinical NLP methods and systems have been developed and showed promising results in various tasks. These methods and tools have also been successfully applied to facilitate clinical research, as well as to support healthcare applications. Recent advancements in artificial intelligence (AI), particularly deep learning-based neural networks, have achieved state-of-the-art performance on diverse NLP tasks in general domain, indicating great opportunities for solving real-world medical problems. At the same time, the amount of health information available online has exploded through use of social media, community forums, and health-related websites. These present additional challenges and opportunities for further development of new NLP methodologies and applications.

## HealthNLP workshop

The goal of this workshop was to provide a unique platform to bring together researchers and practitioners working with health-related free text, and to facilitate close interaction among students, scholars, and industry professionals on health NLP challenges worldwide. We successfully organized the first international workshop on Health Natural Language Processing (HealthNLP 2018) in June, 2018, at New York City, USA [1].. We continued and held the HealthNLP 2019 workshop on June 10th, 2019, at Beijing, China, in conjunction with the IEEE International Conference on Healthcare Informatics (ICHI 2019). The workshop attracted submissions in the form of research papers, poster abstracts, and demonstration papers. All submissions were subjected to rigorous peer-review, with at least two peer-reviews and at least one review by a senior member of the program committee. Selected papers and abstracts were featured as oral / poster presentations at the workshop. We selected and invited eight high-quality submissions to extend their workshop abstracts for this journal supplement.

## Topics

The main focus of the included papers is information extraction from clinical documents using deep learning-based approaches.

Heo et al. [2] proposed a hybrid ranking method that combines a co-occurrence approach considering both direct and indirect entity pair relationship with specialized word embeddings for measuring the relatedness of two entities. They evaluated the proposed ranking method with other well-known methods such as co-occurrence, Word2Vec, COALS, and random indexing by calculating top entities related to Alzheimer’s disease. Furthermore, they conducted analysis of gene, pathway, and gene-phenotype relationships and found that the proposed method could find more hidden relationships than the traditional methods.

Li and Hou [3] integrated the attention mechanism into a neural network, and proposed an improved clinical named entity recognition method for Chinese electronic medical records called BiLSTM-Att-CRF. Medical dictionaries and part-of-speech (POS) features were also introduced. They evaluated the proposed model on China Conference on Knowledge Graph and Semantic Computing (CCKS) 2017 and 2018 Chinese EMRs corpora, and found the model achieved better performance than other widely-used models. Their work preliminarily confirmed the validity of attention mechanism in extracting information from clinical documents.

In Xu et al.’s study [4], they adopted Bidirectional Long Short-Term Memory (BiLSTM) networks and Conditional Random Fields (CRF) to simultaneously identify named entity attributes, and to relate medical concepts to their attributes. Their approach achieved higher accuracy than the traditional systems that tackle two tasks separately on three medical concept-attribute detection tasks: disease-modifier, medication-signature, and lab test-value. They provide a simple yet unified solution to concept-attribute detection without using external data or knowledge bases, and thus streamlined practical clinical NLP systems.

De-identification of clinical notes is one of the most crucial prerequisites for utilizing clinical notes in other downstream biomedical informatics studies. Yang et al. [5] explored de-identification in cross-institute settings using deep learning-based approaches: fine-tuning and pre-training. They pre-trained de-identification models, LSTM-CRF, on the University of Florida (UF) Health corpus and fine-tuned the models on i2b2 datasets. They demonstrated that fine-tuning pre-trained models with a small local corpus (i.e., notes from UF Health) could significantly enhance the performance.

Wang et al. [6] developed and evaluated a rule-based NLP system to capture information on stage, histology, tumor grade and therapies in lung cancer patients using various clinical narrative documents including clinical notes, pathology reports and surgery reports. Their evaluation of the system showed promising results with precisions and recalls for stage, histology, grade, and therapies. They used convolutional neural networks (CNN) in the error analysis, and found that CNN and the proposed NLP system could identify more true labels than the reference standard.

Li et al. [7] developed a disease classification algorithm for accurately recognizing rare diseases from symptom description documents. They leveraged a knowledge graph in representing documents and compared with LSTM models. On two Chinese disease classification data sets, the proposed algorithm delivered robust performance on rare diseases, outperforming a wide range of baselines, including resampling, deep learning, and feature selection methods.

A lack of publicly available clinical corpus resources has become a bottleneck for wide adoption of NLP applications in the clinical domain. Sun et al. [8] demonstrated a Chinese clinical corpus and a novel annotation work for chemical disease semantic extraction. The corpus is chronic disease specific and targeted at combination therapy related mining from biomedical abstracts in Chinese. The result analysis of the corpus verified its quality for the chemical-treat-disease relation identification task. The annotated corpus would be a useful resource for developing useful clinical relation extraction methods and tools.

## Discussion and conclusion

In conclusion, the papers included in this special issue highlight the current research trends in health-related NLP field. With the successful applications of deep learning methods in the general domain, researchers have attempted to apply these methods to medical NLP tasks and have achieved promising results. We envision that these studies will have a significant impact on NLP methodologies, tools, and applications in the healthcare domain.

## Data Availability

Not applicable.

## Abbreviations

AI: artificial intelligence
BiLSTM: Bidirectional Long Short-Term Memory
CCKS: China Conference on Knowledge Graph and Semantic Computing
CNN: convolutional neural networks
CRF: Conditional Random Fields
EHR: electronic health record
HealthNLP: the workshop on Health Natural Language Processing
ICHI: the IEEE International Conference on Healthcare Informatics
NLP: natural language processing
POS: part-of-speech
